# Drying Route Influences Matrix Organization, Reconstitution, Flowability and Selected Phytochemical Indicators of Apricot Powder

**DOI:** 10.3390/foods15142455

**Published:** 2026-07-10

**Authors:** Zhanjun Hu, Hong Zhang, Zhihui Tang, Ruili Zhang

**Affiliations:** 1 College of Mechanical and Electrical Engineering, Tarim University, Alar 843300, China; 2 The Key Laboratory of Utilization and Equipment of Agricultural and Forestry Products, Alar 843300, China; 3 Key Laboratory of Tarim Oasis Agriculture, Tarim University, Ministry of Education, Alar 843300, China; 4 College of Food Science and Engineering, Tarim University, Alar 843300, China

**Keywords:** vacuum freeze drying, vacuum-pulsed drying, matrix organization, powder flowability, reconstitution, selected phytochemical indicators

## Abstract

Apricot powder functionality after drying, grinding and sieving remains insufficiently understood. This study prepared powders from diluted Diaoganxing apricot pulp using hot-air drying (HAD), infrared drying (IRD), vacuum-pulsed drying (VPD) and vacuum freeze drying (VFD), followed by identical grinding and 60-mesh sieving. Moisture status, density, calculated porosity, particle characteristics, reconstitution, flowability, color, selected phytochemical indicators, ferric reducing antioxidant power (FRAP) and supplementary electronic-nose fingerprints were evaluated. The drying route markedly affected powder properties: moisture content and water activity ranged from 7.23 ± 0.38% to 9.85 ± 0.02% and 0.218 ± 0.002 to 0.370 ± 0.007, respectively. VFD gave the highest calculated porosity (59.09 ± 0.84%), shortest wettability time (46.05 ± 1.71 s), highest water-holding capacity (3.63 ± 0.07 g/g), smallest color difference (ΔE = 1.12 ± 0.47), and highest TPC, TCC, AAC and FRAP values. VPD showed the best handling indices, with the lowest angle of repose (28.44 ± 0.34°), Hausner ratio (1.07 ± 0.01) and Carr index (6.33 ± 0.66%). Correlation/PCA indicated treatment-level co-variation, not causality. Under the tested processing conditions, VFD may be preferable for rapid reconstitution and the measured quality indicators, whereas VPD may be more suitable for powder handling.

## 1. Introduction

Apricot (*Prunus armeniaca* L. cv. “Diaoganxing”) is appreciated for its distinctive sensory quality and for its richness in polyphenols, carotenoids, dietary fiber and other nutritionally relevant compounds [1,2]. In apricot-producing regions, however, utilization is still largely dominated by fresh consumption and conventional dried products, which limits product diversification, storage convenience and value addition. Converting apricot pulp into powder is a practical strategy for developing shelf-stable and easy-to-use ingredients for instant beverages, bakery products, composite food systems and convenience-oriented formulations [3,4]. For these applications, product quality depends not only on color and selected composition-related indicators but also on technological attributes such as wettability, water-holding capacity, flowability and physical stability during handling, mixing and packaging [5,6].

Yang et al. [7] compared hot-air drying (HAD), infrared drying (IRD), vacuum-pulsed drying (VPD) and vacuum freeze drying (VFD) for Diaoganxing apricot slices, focusing mainly on drying kinetics, slice microstructure, color, texture, rehydration, nutritional retention, antioxidant activity and energy consumption. That work provides an important basis for selecting drying technologies for dried apricot products. However, the manufacture of apricot powder involves additional processing steps, including pulp dilution, homogenization, thin-layer drying, grinding and sieving. These steps transform the dried matrix into a particulate ingredient, and the resulting powder quality is influenced not only by slice-level attributes but also by powder-specific factors such as particle formation, residual moisture, bulk and tapped density, calculated porosity, particle-size distribution, morphology, wettability, liquid-holding capacity, flowability, compressibility and dispersion behavior. Related powder-engineering research has also shown that formulation changes can substantially influence the physical and aroma-related properties of spray-dried food powders [8]. Therefore, the behavior of dried apricot slices cannot be directly extrapolated to apricot powder used in instant beverages, bakery systems, composite food formulations or industrial mixing and packaging operations.

Drying is a critical unit operation in the manufacture of fruit powders because it directly influences residual moisture status, matrix compactness, particle formation, and the preservation of heat- and oxidation-sensitive compounds. A comparison of five drying methods for kedondong powder likewise showed marked differences in moisture status, wettability, density, flowability and antioxidant activity [9]. Through different heat- and mass-transfer mechanisms, drying environments can modify matrix development and consequently generate different combinations of hydration performance, handling characteristics, and retention-related quality outcomes [10]. Hot-air drying remains widely used due to its operational simplicity and relatively low cost, although prolonged heating often causes shrinkage, structural collapse, and quality loss. Recent work on mango-peel powders further showed that convective drying and freeze-drying produced different drying kinetics, bioactive-compound levels, hygroscopicity, cohesiveness and flowability [11]. Infrared drying can enhance heat-transfer efficiency and shorten the drying process, whereas vacuum-pulsed drying combines reduced-pressure dehydration with intermittent pressure variation, which may help limit structural collapse and facilitate moisture migration. Vacuum freeze drying, involving pre-freezing followed by dehydration under low-pressure and low-oxygen conditions, is generally regarded as favorable for preserving porous structure and sensitive compounds, although its processing cost is considerably higher [12,13,14].

Although a number of studies have examined drying routes for fruit materials, many have focused on isolated quality traits or a limited set of indicators [15,16]. Recent reviews of powdered foods have emphasized the need to consider processing conditions, powder structure, stability and rehydration behavior together [17]. However, fewer studies have linked drying-route-dependent matrix organization, particle formation after grinding and sieving, hydration performance, handling behavior and selected phytochemical-related indicators within the same fruit-powder system. Recent apple-powder research further showed that spray-drying conditions significantly influenced moisture content, porosity, bulk density, dispersibility, solubility, flowability and reconstitution behavior [18]. In this study, “matrix openness” is used only as an interpretive structural descriptor inferred from density, calculated porosity and SEM-based morphology, rather than as an independently quantified causal variable.

Accordingly, this study examined the powder-engineering consequences of HAD, IRD, VPD and VFD for Diaoganxing apricot powder prepared under the same pulp dilution, grinding and 60-mesh sieving procedure. The objective was not to re-evaluate dried apricot slices or to optimize drying kinetics, dryer design, energy consumption or storage stability. Instead, the study compared how route-dependent moisture status, structural descriptors and particle formation were associated with reconstitution, handling, appearance and selected phytochemical-related indicators. This application-oriented comparison aimed to identify trade-offs between powders intended for rapid reconstitution and those intended for blending, conveying, filling and packaging.

## 2. Materials and Methods

### 2.1. Materials

Fresh apricots (*Prunus armeniaca* L. cv. ‘Diaoganxing’) were harvested in July 2025 from Orchard No. 6, Residential Area of Company 13, Regiment 10, Alar City, Xinjiang Uygur Autonomous Region, China. Fruits with uniform maturity, no visible mechanical damage or pest infestation, and similar size (diameter: 2.6 ± 0.2 cm) were selected. The initial moisture content of the fresh apricots was 82.5 ± 0.6%. Before processing, the samples were stored at 4 ± 1 °C for no longer than 7 days and equilibrated at 25 ± 1 °C for 1 h prior to use.

### 2.2. Preparation of Apricot Powder

Apricot powder preparation was carried out through a standardized sequence consisting of raw material pretreatment, pulp homogenization, tray spreading, route-specific drying, grinding, sieving and sealed storage before analysis. The same pulp dilution ratio, homogenization conditions, layer thickness, tray format, sample loading, grinding procedure and sieving size were used for all drying treatments. Therefore, this section describes the common powder-preparation procedure and the route-specific drying conditions used to obtain comparable apricot powder samples for subsequent physicochemical, structural, functional and phytochemical analyses. The powders produced by hot-air drying, infrared drying, vacuum-pulsed drying and vacuum freeze drying were coded as HAD, IRD, VPD and VFD, respectively.

#### 2.2.1. Pulp Preparation

Fresh apricots were washed three times with distilled water and drained. No chemical sterilization or surface disinfection was performed before drying, in order to avoid possible changes in surface composition, color and phytochemical-related indicators. All knives, trays and contact surfaces were cleaned and sanitized before use. The fruits were then manually peeled and pitted, and the flesh was mixed with distilled water at a ratio of 1:1 (*w*/*v*) to obtain a pumpable and uniformly spreadable pulp for tray drying. This dilution was used to standardize feed consistency and tray-spreading behavior among treatments. Therefore, the prepared powders should be interpreted as powders obtained from a standardized diluted apricot pulp matrix rather than from undiluted apricot flesh. The mixture was homogenized using a colloid mill (JMLB-65, Shanghai Kelao Mechanical Equipment Co., Ltd., Shanghai, China) at 2800 rpm to obtain a uniform pulp. The resulting pulp was evenly spread onto stainless-steel trays to a thickness of 8 mm for subsequent drying.

#### 2.2.2. Drying Procedures

Four drying routes were applied under the operating conditions summarized in Table 1. The operating conditions were selected mainly with reference to the drying parameters reported by Yang et al. [7] for Diaoganxing apricot slices and were adjusted according to the requirements of the present pulp-sheet drying and powder-preparation process. These conditions were used to obtain low-moisture dried matrices suitable for grinding and sieving, rather than to conduct a full optimization of each drying technology. HAD was performed using a hot-air dryer/oven (RB-9023A, NANJING RUNBANG MACHINERY TECHNOLOGY CO., LTD, Nanjing, China). IRD was performed using an infrared dryer (QRIR-1.5, fabricated by the College of Engineering, China Agricultural University, Beijing, China). VPD was performed using a custom-built vacuum-pulsed dryer, which was self-designed and fabricated by the School of Mechanical and Electrical Engineering, Tarim University, Alar, China. VFD was performed using a vacuum freeze dryer (L2-06, fabricated by the School of Mechanical and Electrical Engineering, Tarim University, Alar, China). The drying systems were used as controlled processing platforms to generate apricot powders under defined route-specific dehydration conditions. Because the aim of this study was to compare powder attributes rather than dryer design or equipment performance, the drying processes were described using reproducible operating variables, including temperature, vacuum level or pressure-pulsing cycle, infrared radiation intensity, sample layer thickness, sample loading and drying time.

For VFD, the reported 45 °C refers to the final shelf-temperature set point of the freeze-dryer used in the tested protocol and should not be interpreted as the measured product temperature of the apricot matrix throughout drying. The VFD condition was selected mainly with reference to a previously reported protocol for Diaoganxing apricot drying and was adjusted for the present pulp-sheet drying and powder-preparation process. The product temperature, glass-transition/collapse temperature and the transition between primary and secondary drying were not directly monitored in this study. Therefore, the VFD treatment should be interpreted as a literature-based route-specific processing condition used to obtain a grindable low-moisture matrix, rather than as an optimized freeze-drying program for heat-sensitive apricot constituents.

All treatments used the same pulp formulation, tray size, single-layer thickness, sample loading, grinding procedure and sieving size. The stainless-steel trays were 27 cm × 20 cm × 2 cm, and the pulp loading was approximately 432 g per tray, corresponding to approximately 8.0 kg/m^2^. Each treatment was conducted in triplicate using independently prepared batches.

#### 2.2.3. Grinding and Sieving

The dried apricot pulp sheets were ground using a high-speed grinder (RS-FS1406, Royalstar Small Appliance Co., Ltd., Hefei, China) and passed through a 60-mesh sieve (250 μm). For each batch, 100 g of dried apricot sheet was ground using the same high-speed grinder. Grinding was performed intermittently for 15 s per cycle, with cooling intervals between cycles, until the dried sheets could pass through a 60-mesh sieve. The same grinder, sample mass, grinding procedure and sieving size were used for all drying treatments to minimize preparation-related bias. The powder temperature during and immediately after grinding was not monitored, and no cryogenic cooling was applied; therefore, possible frictional heating during high-speed milling cannot be excluded. To ensure comparability among treatments, all dried samples were subjected to the same grinding and sieving procedure. Only the sieved fraction was collected for subsequent analysis. After preparation, the apricot powder samples were sealed in airtight polyethylene bags and stored at 4 °C in the dark. All analyses were completed within 72 h after powder preparation.

### 2.3. Physicochemical and Functional Properties

#### 2.3.1. General Physicochemical Properties

Moisture content was determined by oven drying at 105 °C for 24 h according to the Chinese National Standard GB 5009.3-2016 [19]. Water activity (aw) was measured at 25 ± 1 °C using a water activity meter (HAD-3A, Huake Instrument Co., Ltd., Wuxi, China). Wettability was evaluated as the time required for 1 g of powder to become fully submerged in 100 mL of distilled water at 25 °C [20].

#### 2.3.2. Density and Porosity

Bulk density (*BD*) was determined by placing a known mass of powder into a 10 mL graduated cylinder and recording the occupied volume. Particle density (*PD*) was determined by a pycnometric method according to Jinapong et al. (2008) [21].

(1) $$PD=\frac{weight\;of\;the\;powder}{Total\;volume\;of\;petroleum\;ether\;and\;suspended\;\;particles\left(mL\right)-6}$$

Porosity was calculated from bulk density and particle density using the following equation:

(2) $$\varphi =100-\frac{bulk\;density}{ture\;density}$$

#### 2.3.3. Flowability and Handling-Related Properties

The flow properties of apricot powders were evaluated by the angle of repose, Hausner ratio (*HR*), and Carr index (*CI*). The angle of repose was determined using the fixed-cone method [22]. HR was calculated from tapped density (*TD*) and bulk density (*BD*), and *CI* was calculated using the standard equation.

(3) $$HR=\frac{TD}{BD}$$

(4) $$Carr\;index\;(\%)=\frac{TD-BD}{TD}$$

#### 2.3.4. Color Attributes

Color parameters were measured using a spectrocolorimeter (CR-8, Threenh Technology Co., Ltd., Guangzhou, Guangdong, China) in the CIELAB color system, including L* (lightness), a* (redness/greenness), and b* (yellowness/blueness). Hue angle, chroma, and total color difference (ΔE) were calculated according to the corresponding equations.

(5) Hue angle=tan−1b*a*

(6) $$Chroma=\sqrt{{\left(a^{*}\right)}^{2}+{\left(b^{*}\right)}^{2}}$$

(7) $$∆E=\sqrt{{\left(L_{0}^{*}-L^{*}\right)}^{2}+{\left(a_{0}^{*}-a^{*}\right)}^{2}+{\left(b_{0}^{*}-b^{*}\right)}^{2}}$$

#### 2.3.5. Particle Size and Zeta Potential

Particle-size distribution was determined using a laser particle-size analyzer (Mastersizer 3000, Malvern Instruments, Malvern, Worcestershire, UK). The results were expressed as D(4,3), D_50_, and span. D(4,3) represents the volume-weighted mean diameter, D_50_ represents the median particle diameter, and span was calculated from D_10_, D_50_, and D_90_ to describe the width of the particle-size distribution [23].

(8) $$D(4,3)=\frac{\sum nidi4}{\sum nidi3}$$

(9) $$span=\frac{D_{90}-D_{10}}{D_{50}}$$

Zeta potential was measured using a particle-size analyzer (Litesizer 500, Anton Paar GmbH, Graz, Austria). Powder samples were dispersed in deionized water at a concentration of 0.1 mg/mL, and the resulting suspensions were injected into the instrument for analysis [24]. The pH of the dispersion was not adjusted before zeta-potential measurement, and the equilibrium pH was not recorded during the original experiment. Therefore, the zeta-potential results were interpreted cautiously as auxiliary comparative dispersion indicators obtained under unadjusted aqueous dispersion conditions, rather than as direct evidence of drying-induced surface-charge differences.

#### 2.3.6. Water Holding Capacity (WHC) and Oil Holding Capacity (OHC) Measurements

WHC and OHC were determined according to a previously reported method [25], with slight modifications. Briefly, 0.5 g of apricot powder (*W*_0_) was placed in a centrifuge tube (*W*_1_) containing 10 mL of distilled water or soybean oil and incubated at 25 °C for 30 min. The mixture was then centrifuged at 8000 rpm and 4 °C for 15 min (MGL-16MA, Merrick Instruments Co., Ltd., Shanghai, China). After removing the supernatant, the weight of the centrifuge tube containing the precipitate (*W*_2_) was recorded. WHC and OHC were calculated using the corresponding equation and expressed as g/g.

(10) $$WHC(OHC)=\frac{W_{2}-W_{1}}{W_{0}}$$

### 2.4. Structural Characterization

#### 2.4.1. Surface Morphology

The surface morphology of apricot powders was observed using a scanning electron microscope (JSM-7610F, Hitachi, Tokyo, Japan). Micrographs were obtained at magnifications of 400×, 1000×, and 2000×. Prior to imaging, the samples were coated with platinum to prevent charging [26].

#### 2.4.2. X-Ray Diffraction (XRD)

X-ray diffraction patterns were obtained using an X-ray diffractometer (SmartLab 9 kW X-ray diffractometer, Rigaku Corporation, Akishima, Tokyo, Japan) equipped with a Cu anode X-ray tube. Powder samples were gently pressed into an aluminum sample holder using a glass slide. The diffraction angle ranged from 4° to 40° (2θ), with a scanning rate of 4°/min at 40 kV and 30 mA.

#### 2.4.3. Fourier Transform Infrared Spectroscopy (FTIR)

The functional groups of apricot powders were analyzed using a Fourier transform infrared spectrometer (Nicolet iS50 FTIR spectrometer, Thermo Fisher Scientific Inc., Madison, WI, USA). Powder samples (50 mg, moisture content < 10%, passed through a 60-mesh sieve) were mixed with KBr at a ratio of 1:100 (mg/mg), compressed into transparent tablets, and scanned in the range of 4000–400 cm^−1^ with a resolution of 4 cm^−1^ over 32 scans.

The term “matrix openness” is defined in the Introduction and was interpreted using density, calculated porosity and SEM observations rather than as an independently measured variable. Specific surface area, surface roughness, pore connectivity and accessible pore volume were not quantitatively measured and were therefore considered only as possible contributors to the inferred structural accessibility.

### 2.5. Supplementary Electronic-Nose Aroma Fingerprinting

Electronic-nose analysis was used as a supplementary aroma-fingerprinting tool to compare the overall sensor-response patterns of apricot powders produced by different drying routes, following the general approach used for dried fruit aroma fingerprinting [27]. Briefly, 2 g of powder was placed in a 35 mL sealed vial and equilibrated at 25 °C for 30 min. Measurements were performed using a PEN3.5 electronic nose (AIRSENSE Analytics GmbH, Schwerin, Germany). The sensor cleaning time, sample detection time, internal flow rate and injection flow rate were set to 600 s, 60 s, 400 mL/min and 400 mL/min, respectively. Data were collected and processed using WinMuster V2.0 software. The response characteristics of the 10 sensors are provided in Table A1. Principal component analysis (PCA) was used to visualize differences in the overall sensor-response fingerprints among drying treatments and to examine the sensor-loading patterns associated with treatment separation. For sensor-pattern interpretation and PCA, the mean response value of each sensor during the stable measurement period of 51–60 s was used as the representative response of each sample.

### 2.6. Phytochemical Indicator Assays and Ferric Reducing Antioxidant Power (FRAP) Assay

Unless otherwise stated, phytochemical-related assays were performed using freshly prepared extracts obtained separately for each analytical method under the extraction conditions described below. For each independently prepared sample batch, extractions and measurements were conducted in triplicate, and the resulting values were averaged as the batch-level value. Treatment means and standard deviations were then calculated from the three independent batches.

#### 2.6.1. Total Phenolic Content (TPC)

TPC was determined using the Folin–Ciocalteu colorimetric method with slight modifications [28]. Approximately 0.1 g of sample powder was extracted with 2.5 mL of 60% ethanol by ultrasonic treatment at 300 W with 5 s pulse and an 8 s interval at 60 °C for 30 min. The extract was centrifuged at 12,000 rpm and 25 °C for 10 min, and the supernatant was adjusted to 2.5 mL with the extraction solvent. Briefly, 0.5 mL of appropriately diluted apricot powder extract was mixed with 2.5 mL of 10% (*v*/*v*) Folin–Ciocalteu reagent. After 5 min, 2 mL of 7.5% (*w*/*v*) sodium carbonate solution was added. The mixture was incubated in the dark at room temperature for 30 min, and the absorbance was measured at 760 nm using a microplate reader (Rayto RT-6100, Rayto Life and Analytical Sciences Co., Ltd., Shenzhen, China). Results were calculated using a tannic acid standard curve and expressed as mg tannic acid equivalent per g dry powder (mg TAE/g).

#### 2.6.2. Total Sugar and Reducing Sugar Contents

Total sugar content (TSC) and reducing sugar content (RSC) were determined using the 3,5-dinitrosalicylic acid (DNS) method [29]. For TSC determination, 0.1 g of powder was hydrolyzed with 2 mL of 6 M HCl in a boiling water bath for 30 min. The hydrolysate was then neutralized with NaOH, diluted to 10 mL with distilled water, and centrifuged, and the supernatant was used for analysis. For RSC determination, 0.2 g of powder was extracted with distilled water at 50 °C for 20 min, followed by centrifugation, and the supernatant was collected. In both assays, the sample extract or glucose standard solution was mixed with DNS reagent, heated in a boiling water bath for 5 min, and cooled to room temperature, and the absorbance was measured at 540 nm. Results were expressed as mg glucose equivalent per g dry powder (mg/g).

#### 2.6.3. Total Carotenoid Content (TCC)

Total carotenoid content was determined using a commercial carotenoid assay kit based on a visible spectrophotometric method according to the manufacturer’s instructions (ZC-S0863, microplate method; Shanghai Zci Bio Technology Co., Ltd., Shanghai, China), with slight modifications. Briefly, 0.100 g of apricot powder was accurately weighed and placed in a pre-cooled mortar. Then, 1.0 mL of distilled water and approximately 10 mg of Reagent 1 were added, and the sample was thoroughly ground under dim light. Reagent 1 was supplied with the carotenoid assay kit and used according to the manufacturer’s instructions. The homogenate was transferred into a 10 mL centrifuge tube. The mortar and pestle were rinsed sequentially with 3.0, 3.0, and 2.0 mL of 80% acetone, and all rinsing solutions were combined in the same centrifuge tube. The extract was then brought to a final volume of 10.0 mL with 80% acetone. The tube was wrapped with aluminum foil and kept in the dark for 3 h, during which the mixture was inverted twice to ensure complete extraction. The extraction was considered complete when the residual tissue became nearly colorless. If visible residues were present in the upper extract, 1.2 mL of the extract was centrifuged at 4000 rpm for 5 min at room temperature. Subsequently, 1.0 mL of the supernatant was transferred into a 1 cm glass cuvette, and the absorbance was measured at 440 nm using 80% acetone as the blank. Each sample was extracted and analyzed in triplicate. Total carotenoid content was calculated according to the manufacturer’s instructions and expressed as mg/g dry powder.

#### 2.6.4. Ascorbic Acid Content (AAC)

Ascorbic acid content was measured using a commercial assay kit based on the Fast Blue Salt B colorimetric method according to the manufacturer’s instructions. Reagents 1–3 were supplied with the kit (ZC-S0336, microplate method; Shanghai Zci Bio Technology Co., Ltd., Shanghai, China). Briefly, approximately 0.1 g of powder was mixed with the extraction solution at a sample-to-solvent ratio of 1:5 (g/mL), homogenized in an ice bath, and centrifuged at 8000× *g* and 4 °C for 20 min. Then, 100 μL of supernatant was mixed with Reagent 1 (30 μL), Reagent 2 (50 μL), Reagent 3 (120 μL), and distilled water (700 μL). After incubation at 25 °C for 20 min, the absorbance was measured at 420 nm using a microplate reader (Rayto RT-6100, Rayto Life and Analytical Sciences Co., Ltd., Shenzhen, China). Results were expressed as μg/g dry powder.

#### 2.6.5. FRAP Assay

Antioxidant capacity was evaluated using the ferric reducing antioxidant power (FRAP) method with a commercial kit (ZC-S0354, microplate method; Shanghai Zci Bio Technology Co., Ltd., Shanghai, China). Briefly, 0.1 g of powder was mixed with 1.0 mL of pre-cooled extraction solution, homogenized, and centrifuged at 10,000 r/min for 5 min at 4 °C to obtain the supernatant. A standard curve was prepared using an FeSO_4_ standard solution, and absorbance was measured at 593 nm according to the kit instructions. Antioxidant capacity was expressed as U g^−1^ dry weight, where one unit (U) was defined as the antioxidant capacity equivalent to 1 μmol of Fe^2+^ standard producing the same absorbance change at 593 nm under the assay conditions.

### 2.7. Statistical Analysis

Each drying treatment was performed using three independently prepared sample batches. Unless otherwise stated, each analytical measurement was conducted for each independent batch, and the results are expressed as mean ± standard deviation (SD, *n* = 3). Prior to ANOVA, normality and homogeneity of variance were checked using the Shapiro–Wilk test and Levene’s test, respectively. Data satisfying these assumptions were analyzed by one-way analysis of variance (ANOVA), and mean comparisons were performed using Duncan’s multiple range test at *p* < 0.05. Pearson correlation analysis was performed using replicate-level data from independently prepared batches (*n* = 12; four drying treatments × three independent batches). Because these data were clustered within only four treatment groups and several variables changed simultaneously across drying routes, the correlations were used only for exploratory pattern recognition and should not be interpreted as evidence of mediation or direct causal pathways. Principal component analysis (PCA) was performed using the same replicate-level dataset after z-standardization of the variables included in the PCA biplot, namely bulk density, particle density, calculated porosity, D50, wettability time, WHC, OHC, angle of repose, Carr index, TPC and FRAP. The PCA biplot was used to visualize treatment-level multivariate separation and the directions of variable loading vectors. All statistical analyses and data-based figures were prepared using OriginPro 2025 (OriginLab Corporation, Northampton, MA, USA; https://www.originlab.com/), except for electronic-nose data processing, which was performed using WinMuster V2.0 software.

## 3. Results and Discussion

### 3.1. Drying Route Differentiated End-Point Moisture and Matrix Compactness

The drying route had a clear influence on the end-point moisture status of apricot powder (Table 2). In the obtained powders, the moisture content decreased from 9.85 ± 0.02% in HAD powder to 7.23 ± 0.38% in VFD powder, and water activity followed the same general trend, decreasing from 0.370 ± 0.007 to 0.218 ± 0.002. From a product-quality and shelf-stability perspective, water activity is more informative than moisture content because it reflects the amount of water available for microbial growth and moisture-dependent deterioration reactions. The U.S. Food and Drug Administration uses aw = 0.85 as an important threshold for water-activity-controlled foods [30]. In the present study, all apricot powders had aw values far below both 0.85 and 0.60, indicating that the freshly prepared powders were within a low-water-activity range unfavorable for microbial growth under the tested conditions [31,32]. Nevertheless, a low aw should not be interpreted as sterilization or as direct evidence of long-term storage stability, because microorganisms may survive in low-moisture foods and moisture uptake may still occur during grinding, sieving, packaging or storage. Therefore, hygienic processing, moisture-barrier packaging and storage-condition control remain necessary for future industrial application.

The drying route also significantly affected density-related properties. HAD showed the highest bulk density, whereas VFD showed the lowest bulk density, tapped density and particle density. These results indicate that the drying treatments differed not only in residual moisture status but also in density-related and calculated porosity characteristics of the resulting powders. The lower density values observed for VPD and VFD were consistent with the formation of less compact powder structures than those obtained by atmospheric thermal drying. This interpretation was also consistent with the calculated porosity values, among which VFD showed the highest value.

The density and porosity results further indicate that the drying route changed the mechanical state of the dried matrix before grinding. HAD produced the highest bulk density (0.56 ± 0.01 g/cm^3^) and particle density (1.21 ± 0.02 g/cm^3^), which may be related to cell shrinkage, capillary contraction and matrix collapse during prolonged atmospheric heating. In contrast, VFD produced the lowest bulk density (0.46 ± 0.01 g/cm^3^), the lowest particle density (1.12 ± 0.01 g/cm^3^) and the highest calculated porosity (59.09 ± 0.84%). Together with the SEM observations, these measurements indicate a lighter and less compact VFD matrix; however, pore connectivity and accessible pore volume were not directly measured. This difference is important for powder-product design because a porous structure can facilitate water penetration during reconstitution, whereas a compact structure may slow wetting and dispersion.

Wettability varied consistently with these structural differences. VFD required the shortest time for complete wetting, whereas HAD showed the slowest wetting behavior. For instant beverage or reconstituted fruit-powder applications, this difference is relevant because wettability determines how rapidly powder particles are penetrated by water before stirring or mixing. However, water ingress may also be influenced by residual moisture, particle-size distribution, particle morphology, surface composition and capillary characteristics [33]. Thus, the wettability differences reflected the combined effects of residual moisture status, density-related structure, particle formation and drying-induced surface or capillary characteristics, rather than porosity alone.

Taken together, these results indicate that the drying route differentiated apricot powder in terms of both moisture-related status and matrix compactness. Because matrix compactness can influence fracture behavior during grinding and powder packing characteristics, these structural differences were expected to contribute to subsequent variation in particle attributes and handling performance [34]. The later functional and phytochemical-related differences should therefore be interpreted as treatment-level outcomes influenced jointly by the drying environment, residual moisture status, matrix organization and particle formation.

### 3.2. Drying Route Differentiates Particle Formation and Handling-Related Behavior

**Figure 1:**
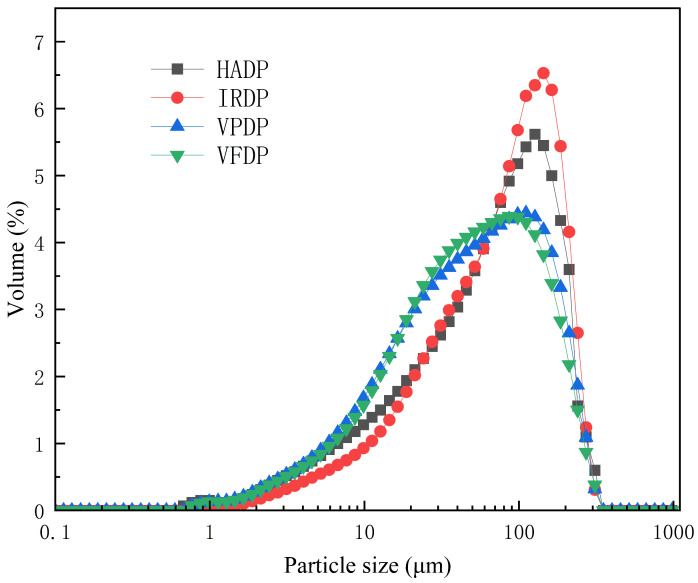
Particle-size distributions of apricot powders produced by different drying routes.

The drying route significantly influenced particle characteristics of apricot powder (Table 2; Figure 1). HAD and IRD generally produced coarser powders than the vacuum-based treatments, whereas VPD and VFD yielded smaller median particle sizes. At the same time, the vacuum-based treatments showed broader particle-size distributions, as reflected by their larger span values. Because all treatments were subjected to the same grinding and sieving procedure, the smaller median particle sizes of VPD and VFD were consistent with treatment-dependent differences in fracture behavior. However, brittleness and fracture resistance were not directly measured, and residual moisture, local structural heterogeneity and milling-related breakage may also have contributed. In contrast, IRD showed the narrowest span, indicating comparatively better particle-size uniformity under the present conditions.

The numerical particle-size results further support this interpretation. D50 decreased from 80.70 ± 1.11 μm in HAD powder and 86.33 ± 0.61 μm in IRD powder to 55.17 ± 0.64 μm in VPD powder and 52.87 ± 0.84 μm in VFD powder. From a conventional powder-flow perspective, this reduction in D50 would normally be expected to increase the interparticle contact area, cohesion and friction, thereby reducing flowability. Therefore, the superior flowability of VPD powder cannot be explained by median particle size alone. Because all treatments were subjected to the same grinding and sieving procedure, the smaller D50 values of VPD and VFD were consistent with the route-dependent fracture behavior of the dried matrices. However, fracture behavior may also have been influenced by residual moisture, structural heterogeneity and milling-related breakage. The larger span values of VPD and VFD further indicate that the smaller median particle sizes were accompanied by broader particle-size distributions rather than greater particle-size uniformity.

The differences in particle formation were accompanied by pronounced differences in handling-related characteristics (Table 3). Among all treatments, VPD exhibited the lowest angle of repose, Hausner ratio, and Carr index, indicating superior handling performance. In contrast, VFD showed the largest angle of repose, although its Hausner ratio and Carr index remained lower than those of HAD. This result suggests that greater matrix openness does not necessarily result in optimal free-flow behavior. While a porous and low-density structure may facilitate rapid reconstitution, it may also intensify interparticle friction, cohesion, or mechanical interlocking, thereby impairing powder flow. Because particle sphericity, circularity, the aspect ratio, surface roughness and cohesive forces were not quantitatively measured, the proposed explanation remains qualitative. Future work should combine SEM image analysis, shear-cell testing and moisture-controlled flow measurements to verify the relative contributions of particle morphology and capillary cohesion [35,36].

The best flowability of VPD was likely associated with a favorable combination of external particle morphology, particle-size distribution, packing behavior and residual moisture status. Based on SEM images, VPD particles showed relatively distinct lamellar fracture features and less severe surface collapse than HAD, while appearing less fluffy and less mechanically interlocking than VFD particles. In addition, the lower water activity of VPD powder (aw = 0.270) compared with HAD powder (aw = 0.370) likely minimized capillary condensation and liquid-bridge formation, thereby reducing interparticle cohesion and contributing to its lower angle of repose. This moisture-related effect is particularly relevant for amorphous sugar-rich fruit powders, because local moisture can plasticize the matrix, lower the glass transition temperature (Tg), and increase stickiness or caking even when the overall aw remains low. By contrast, VFD showed the highest porosity and shortest wettability time but the largest angle of repose, indicating that structural features favorable for water penetration can coincide with greater surface roughness, irregular particle shape or mechanical interlocking. Therefore, reconstitution and handling should be considered related but distinct quality dimensions.

All apricot powder dispersions showed negative zeta-potential values under the tested aqueous dispersion conditions. However, because the dispersions were not adjusted to a fixed pH and the equilibrium pH was not recorded during the original measurements, the zeta-potential differences should not be interpreted as direct evidence that the drying route alone modified particle surface charge. Instead, these values are presented only as auxiliary comparative dispersion indicators. Future studies should record the equilibrium pH of powder dispersions or conduct zeta-potential measurements under controlled pH conditions to better distinguish surface-charge effects from pH-dependent ionization of soluble acids, pectin-related groups and other dissolved constituents.

Overall, the particle-size and flowability results show that the powders produced by the four drying treatments differed in their apparent fracture outcomes after standardized grinding and in their subsequent handling behavior. Importantly, handling-oriented powder quality followed a differentiation pattern distinct from calculated porosity or inferred matrix openness. VFD showed the shortest wettability time together with the highest calculated porosity, whereas VPD showed the lowest angle of repose, Hausner ratio and Carr index. These results indicate different application-related advantages, but they do not demonstrate that porosity alone caused the reconstitution differences or that the drying route alone controlled flowability. This distinction is important for apricot powder-product design: powders intended for instant beverages should prioritize wetting and hydration properties, whereas powders intended for blending, conveying, filling and packaging should prioritize flowability and low compressibility.

### 3.3. Structural and Moisture-Related Characteristics Were Associated with Hydration Functionality

Hydration-related properties differed significantly among drying treatments and followed the combined trends in moisture status, density, calculated porosity and particle characteristics (Table 2). Powders obtained by vacuum-based drying, particularly VFD, exhibited shorter wettability times and higher water-holding capacities than those produced by atmospheric thermal drying. VFD also showed the greatest oil-holding capacity, although OHC differed less markedly than WHC. The simultaneous decrease in density and increase in calculated porosity suggests that a less compact matrix favored liquid penetration and retention [37]. However, residual moisture, particle size, morphology, surface chemistry and milling-induced breakage changed together; therefore, the shorter wettability time of VFD is best regarded as a treatment-level outcome rather than an effect of porosity alone.

**Figure 2:**
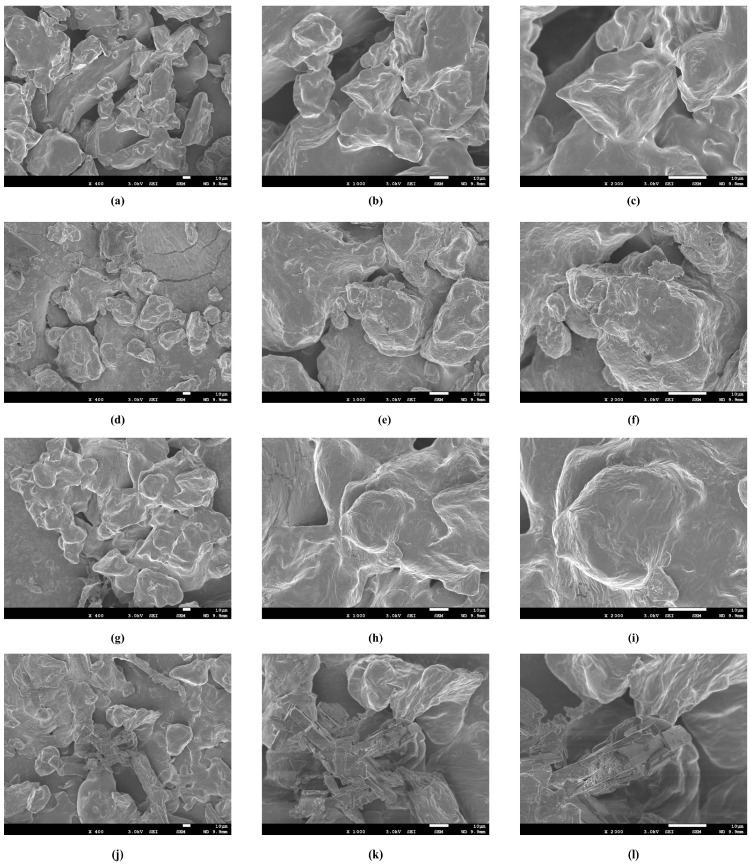
Scanning electron micrographs of apricot powders produced by different drying routes: HAD at 400× (**a**), 1000× (**b**), and 2000× (**c**); IRD at 400× (**d**), 1000× (**e**), and 2000× (**f**); VPD at 400× (**g**), 1000× (**h**), and 2000× (**i**); and VFD at 400× (**j**), 1000× (**k**), and 2000× (**l**).

SEM observations supported this interpretation and also provided qualitative evidence for the differences in flowability among powders (Figure 2). HAD generated relatively dense and collapsed particles with evident shrinkage and contraction, whereas IRD produced rougher surfaces accompanied by more noticeable cracking and localized agglomeration. VPD showed more distinct lamellar fracture features and less visually apparent collapse, which may have favored particle rearrangement and contributed to its lower angle of repose, Hausner ratio and Carr index. In contrast, VFD exhibited a comparatively open and less compact morphology, but its particles appeared more irregular and prone to mechanical interlocking, which may explain why VFD showed excellent wettability but not the best flowability. Previous studies have also reported that drying-induced changes in particle morphology, pore structure and surface characteristics can strongly influence powder hydration and handling behavior [38]. Thus, the SEM observations support differences in both matrix accessibility and external particle morphology relevant to powder flow. Although a less compact morphology could provide more accessible pathways for liquid penetration, direct measurements of specific surface area, surface roughness, pore connectivity, accessible pore volume, capillary dimensions and adsorption-site density are still needed to verify this mechanism.

XRD and FTIR were retained as supplementary structural characterization tools, and the corresponding patterns are provided as Figures S1 and S2. Because these analyses did not directly quantify pore connectivity, accessible pore volume or interparticle forces, they were used only as supportive evidence of treatment-dependent structural differences.

The hydration results also highlight an important practical distinction: the structural features that favor rapid reconstitution are not necessarily those that favor good powder handling. Under the present conditions, VFD was most advantageous for hydration-related functionality, whereas VPD performed better in terms of handling-related indices. This distinction is relevant to ingredient design because powders used in instant beverages may require rapid wetting and high water uptake, whereas powders intended for industrial blending, packaging and conveying may require better flowability and lower compressibility. Overall, the structural analyses showed that the drying treatments produced differences in morphology, density, calculated porosity and short-range organization. Their co-variation with wettability and liquid-holding capacity supports a structural contribution to hydration behavior, but the relative effects of residual moisture, particle size and surface characteristics cannot be separated from the present data.

### 3.4. Drying Route Influenced Appearance and Selected Phytochemical Indicators

The drying route markedly influenced the appearance-related quality of apricot powder (Table 4). VFD showed the smallest total color difference relative to the fresh homogenized apricot pulp before drying and retained L*, b*, chroma and hue angle values closest to those of the pre-drying pulp, indicating superior visual preservation. In contrast, HAD resulted in the greatest color deterioration, whereas IRD and VPD showed intermediate performance. This pattern was likely related to lower oxygen exposure and the protective effects associated with pre-freezing and low-pressure dehydration in VFD. Because product temperature was not monitored during freeze drying, these color and phytochemical-related advantages represent outcomes of the complete tested VFD protocol rather than direct evidence of uniformly low thermal exposure.

**Figure 3:**
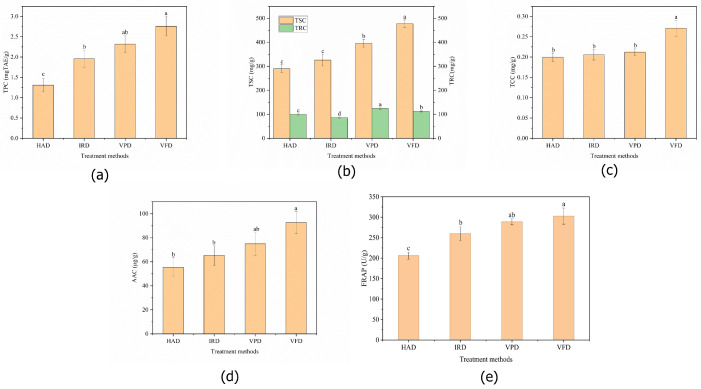
Phytochemical-related indicators and ferric reducing antioxidant power (FRAP) values of apricot powders produced by different drying routes: total phenolic content (**a**), total sugar and reducing sugar contents (**b**), total carotenoid content (**c**), ascorbic acid content (**d**), and FRAP values (**e**). Different lowercase letters indicate significant differences among drying treatments within the same indicator (*p* < 0.05).

A comparable pattern was observed for the selected phytochemical-related indicators (Figure 3). VFD showed the highest measured TPC, TCC, AAC and FRAP values, while HAD and IRD generally showed lower values [39]. VPD also showed higher values than the atmospheric thermal routes for several measured indicators, including total phenolics and total sugars. Similar evidence from spray-dried pumpkin powder indicates that processing conditions can markedly influence moisture-related properties, particle characteristics, color, oil absorption capacity, phenolic content, carotenoid-related compounds and antioxidant capacity, highlighting the need to evaluate physical functionality and bioactive-related quality together [40]. Overall, VFD gave more favorable values for the measured TPC, TCC, AAC and FRAP indicators, but these data do not demonstrate superior preservation of the complete apricot phytochemical profile. The measured values reflect both compound retention and extractability under the specified extraction conditions, while the relative effects of oxygen exposure, drying history, residual moisture and milling-induced heating could not be separated [41].

**Figure 4:**
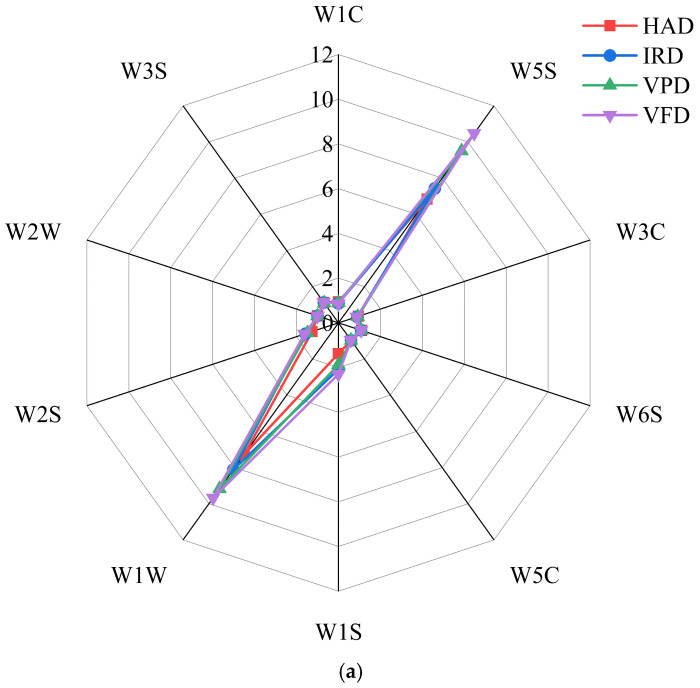

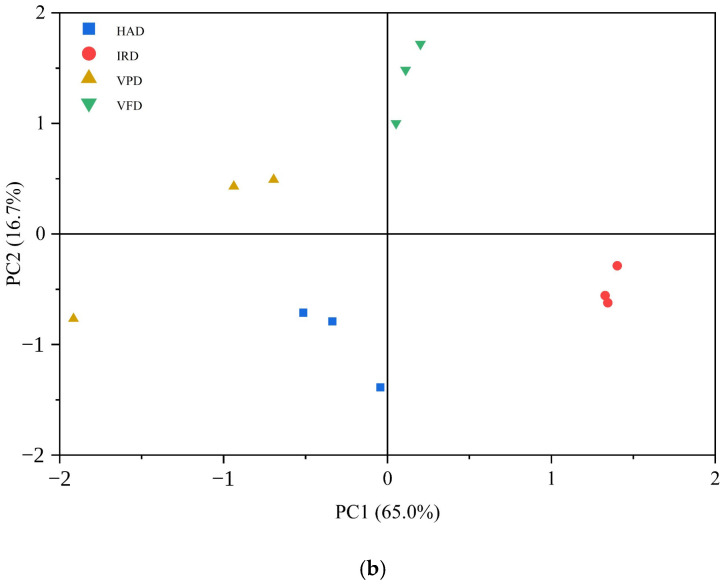
Supplementary electronic-nose aroma fingerprints of apricot powders produced by different drying routes: (**a**) radar chart of overall sensor responses; (**b**) PCA score plot. Sensor response characteristics are listed in Table A1.

Electronic-nose analysis was used as a supplementary aroma-fingerprinting approach rather than as a compound-identification method. The radar and PCA plots showed that the overall electronic-nose response patterns differed among powders produced by different drying routes (Figure 4). To further identify the sensor groups associated with electronic-nose differentiation, PCA was performed using the standardized mean sensor responses during the stable measurement period of 51–60 s. PC1 and PC2 explained 65.0% and 16.7% of the total variance, respectively, accounting for 81.7% of the cumulative variance. The PCA loading results showed that PC1 was mainly associated with positive loadings of W1S, W2S, W1W and W5S, whereas PC2 was mainly associated with W3S and W2W. According to Table A1, W5S is a broad-range sensor, W1W is associated with sulfides, W1S is sensitive to methyl-related responses, W2S responds broadly to alcohols, aldehydes and ketones, W3S is related to long-chain alkanes, and W2W is sensitive to organic sulfides. Therefore, the treatment-dependent electronic-nose separation was mainly related to broad-range sensor responses, sulfide-related responses, methyl-related responses and alcohol/aldehyde/ketone-related responses, with additional contributions from long-chain alkane- and organic sulfide-related responses. However, because the electronic nose provides cross-sensitive sensor-response fingerprints rather than compound-level volatile identification, these results should be interpreted only as supplementary evidence of aroma-pattern differentiation rather than as direct identification of specific volatile compounds or evidence of superior aroma preservation. Compound-level volatile analysis by GC–MS or GC–IMS, together with sensory evaluation, would be useful in future studies to identify the specific volatile compounds and sensory relevance of these differences.

In summary, the powders differed in physical functionality, color attributes and the selected TPC, TCC, AAC and FRAP indicators. Because multiple structural, functional and phytochemical-related indicators changed simultaneously across treatments, correlation analysis was further used to clarify the relationships among these responses within the process–matrix–powder-function framework.

### 3.5. Exploratory Correlation and PCA Analyses Highlighted Linked and Divergent Quality Dimensions

**Figure 5:**
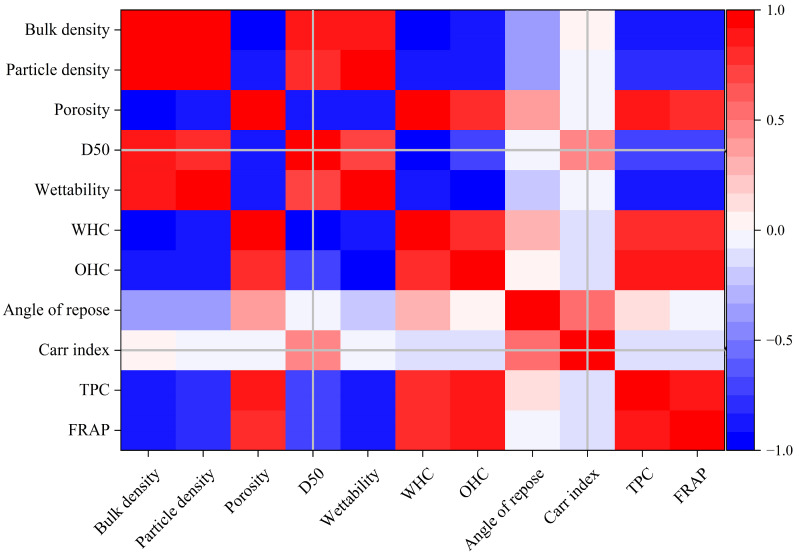
Exploratory Pearson correlation heatmap of structural, functional and phytochemical-related indicators of apricot powders. The heatmap describes treatment-level co-variation based on four drying treatments with three independent batches per treatment and should not be interpreted as evidence of direct causal relationships.

To explore treatment-level co-variation among structural attributes, powder functionality and selected phytochemical-related indicators, Pearson correlation analysis was carried out as an exploratory analysis (Figure 5). Because the dataset consisted of four drying treatments with three independent batches per treatment, the resulting correlations may reflect drying-route group effects and should not be interpreted as direct bivariate causal relationships. In the present dataset, calculated porosity was negatively associated with wettability time and positively associated with WHC and OHC, whereas bulk density, particle density and D50 tended to show the opposite relationships. These associations indicate that the measured structural descriptors co-varied with reconstitution-related behavior across treatments, but they cannot distinguish the independent effects of porosity, residual moisture, particle morphology or grinding-related fracture behavior.

The correlation results also showed associations between the measured structural descriptors and selected phytochemical-related indicators. Porosity, WHC and OHC showed positive associations with TPC and FRAP-related reducing capacity, whereas compactness-related variables showed the opposite tendency. However, this association was likely influenced by the VFD group, which simultaneously showed high calculated porosity and high measured TPC and FRAP values. Therefore, the positive relationship between porosity and phenolic-related reducing capacity should be interpreted as treatment-level co-variation rather than as evidence that porosity directly improved phenolic retention or reducing capacity. Residual moisture status, oxygen exposure, thermal history and extractability changed simultaneously across drying treatments and could not be separated by the present correlation analysis.

**Figure 6:**
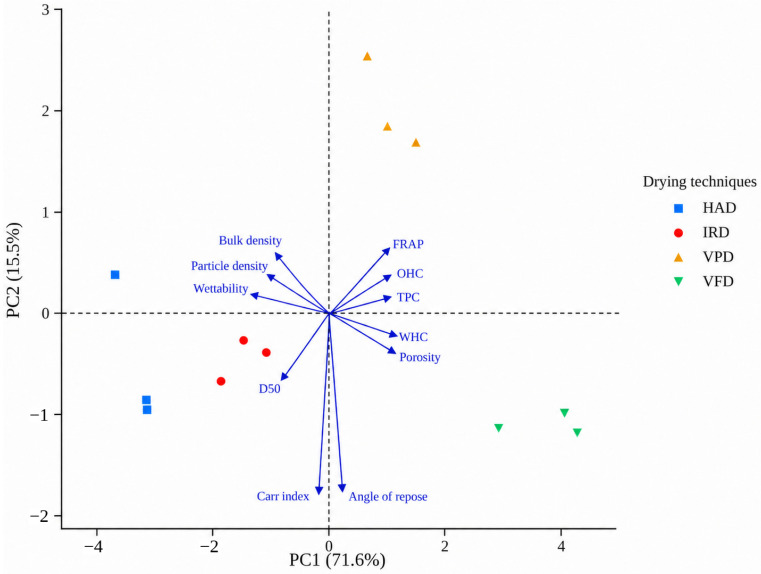
Principal component analysis (PCA) biplot of standardized structural, hydration-related, flowability-related, and phytochemical-related indicators of apricot powders produced by hot-air drying (HAD), infrared drying (IRD), vacuum-pulsed drying (VPD), and vacuum freeze drying (VFD). Points represent the three independently prepared batches for each drying route, arrows represent variable loading vectors, and dashed lines indicate the PC1 = 0 and PC2 = 0 reference axes. PC1 and PC2 explained 71.6% and 15.5% of the total variance, respectively. All variables were z-standardized before PCA.

To complement the exploratory pairwise correlation analysis, PCA was performed to visualize treatment-level multivariate patterns among apricot powders produced by different drying routes (Figure 6). PC1 and PC2 explained 71.60% and 15.51% of the total variance, respectively, accounting for 87.11% of the cumulative variance. The PCA biplot showed a clear separation among the four drying treatments, indicating coordinated differences in multiple powder attributes.

VFD samples were located in the direction of higher calculated porosity, WHC, OHC, TPC, and FRAP. In contrast, HAD samples were positioned in the direction of higher bulk density, particle density, and wettability time, whereas IRD samples were located closer to the D50 vector. VPD samples were separated primarily along the positive PC2 direction and showed an orientation opposite to the angle of repose and Carr index vectors, consistent with its favorable handling-related properties.

The PCA biplot further supported the distinction between hydration-related and handling-related quality dimensions. Hydration-related and selected phytochemical-related variables tended to co-vary with calculated porosity, whereas compactness-related variables and wettability time were oriented in the opposite direction. Flowability-related variables showed a different directional pattern, indicating that powder handling was not governed by the same variable set as rapid reconstitution. These PCA patterns should be interpreted as treatment-level multivariate co-variation because drying route, residual moisture status, matrix organization, particle formation and extractability-related factors changed simultaneously across treatments.

Overall, the results can be organized using an application-oriented process–matrix–powder–function framework. The framework is intended as an integrative decision-support approach rather than as a validated causal model. Its practical value lies in linking measured powder properties with different end-use requirements. VFD is more appropriate when rapid reconstitution, color retention and selected phytochemical-related indicators are prioritized, whereas VPD is more appropriate when powder handling, mixing, conveying and packaging are the main processing requirements. Verification of the proposed mechanisms would require moisture-controlled experiments and direct measurements of pore connectivity, particle shape, surface roughness, interparticle forces and fracture behavior.

### 3.6. Practical Implications and Limitations

The present results should be interpreted at the powder-ingredient level rather than at the dried-slice level. Although the same apricot cultivar and similar drying-route categories have been investigated previously for dried apricot slices, powder production introduces a further transformation step in which the dried matrix is fractured, packed, dispersed and rehydrated as a particulate material. Therefore, quality attributes such as density, porosity, particle-size distribution, wettability, water-holding capacity, oil-holding capacity, the Hausner ratio, the Carr index and the angle of repose become central to product performance. In this context, VFD showed the lowest density, highest calculated porosity, shortest wettability time and highest water-holding capacity, whereas VPD produced the most favorable handling-related indices. This divergence indicates that the “best” drying route depends on whether the powder is designed for instant reconstitution or for industrial handling, mixing and packaging.

The results provide a practical basis for selecting drying routes according to the intended use of apricot powder. VFD showed the most favorable reconstitution behavior, the smallest color change and the highest values of the selected phytochemical-related indicators, suggesting its potential suitability for high-value applications in which these quality attributes are prioritized. However, these advantages should be balanced against the relatively long processing cycle, greater equipment complexity and higher processing cost associated with freeze drying. Under the present experimental conditions, VFD included a 24 h pre-freezing step and the longest listed drying time of 48 h, which may limit production throughput and increase refrigeration and vacuum-operation requirements during scale-up. VPD, although not producing the highest phytochemical-related values, gave the best flowability and handling-related indices, indicating its potential suitability for powder systems that require conveying, mixing, packaging or incorporation into composite food formulations. HAD and IRD may remain relevant as operationally simpler thermal routes, but their implementation should be balanced against the greater color changes and lower values of several measured quality indicators observed under the present conditions. Because energy consumption, equipment investment, labor demand, product yield and unit production cost were not quantified, this industrial comparison should be regarded as qualitative rather than as a techno-economic ranking.

Several limitations should be considered when interpreting the results. First, the powders were compared under low-moisture but not identical end-point moisture conditions; therefore, the observed differences reflect the combined effects of drying route, final moisture status, matrix organization and particle formation rather than a moisture-controlled mechanistic comparison. Second, zeta-potential values were obtained under unadjusted aqueous dispersion conditions, and equilibrium pH was not recorded; therefore, they were interpreted only as auxiliary comparative dispersion indicators. Third, electronic-nose analysis described global sensor-response patterns rather than compound-level volatile profiles. Fourth, the powder temperature during high-speed grinding was not monitored, which may affect heat-sensitive compounds and amorphous matrix behavior during milling. Fifth, the 1:1 pulp dilution used to standardize feed consistency and tray spreading may have altered the effective concentration and distribution of low-molecular-weight sugars, such as fructose and glucose, which can act as plasticizers in amorphous fruit powders. Although this dilution improved experimental comparability, industrial formulators should account for this dilution factor when predicting the glass transition temperature (Tg), stickiness, caking risk, storage stability and final powder dosage. Future studies should include temperature-controlled milling, undiluted or concentrated pulp systems, controlled-pH dispersion analysis, compound-level volatile profiling, hygroscopicity and storage-stability evaluation, and pilot-scale energy and cost assessment [42].

## 4. Conclusions

The four drying treatments produced significant differences in moisture status, structural descriptors, powder functionality, color and selected phytochemical-related indicators of Diaoganxing apricot powder under the tested processing conditions. Because the treatments differed in final moisture content and water activity, the measured differences cannot be assigned exclusively to drying technology or inferred matrix openness. VFD showed the lowest density, the highest calculated porosity, the shortest wettability time, the highest water-holding capacity, the smallest color difference and the highest values of the selected phytochemical indicators. In contrast, VPD showed the best powder handling performance, as indicated by the lowest angle of repose, Hausner ratio and Carr index. Exploratory correlation and PCA analyses indicated treatment-level co-variation among structural, functional and phytochemical-related indicators, but these associations do not establish direct causal relationships. Overall, drying-route selection for apricot powder should be application-oriented. VFD may be preferable when rapid reconstitution, small color change and higher values of the measured phytochemical indicators are prioritized, whereas VPD may be more suitable when flowability, mixing, conveying and packaging performance are the major requirements. However, the quality advantages of VFD should be balanced against its longer processing cycle, greater equipment requirements and higher processing cost. Further moisture-controlled experiments, storage-stability evaluation, pilot-scale techno-economic assessment, controlled-pH zeta-potential measurements and temperature-controlled milling studies are required before the industrial feasibility of the four drying routes can be directly compared.

## Figures and Tables

**Table 1:** Operating conditions used for apricot powder preparation by different drying routes.

Drying Route	Main Operating Conditions	Drying Time
Hot-air drying (HAD)	60 °C; air velocity, 1.5 m/s	28 h
Infrared drying (IRD)	60 °C; infrared intensity, 1.1 kW/m^2^; wavelength, 5–15 μm	25 h
Vacuum-pulsed drying (VPD)	60 °C; vacuum degree, 6 kPa; pressure cycle, 3 min atmospheric pressure followed by 12 min vacuum	20 h
Vacuum freeze drying (VFD)	Pre-freezing at −20 °C for 24 h; condenser temperature, −40 °C; final shelf-temperature set point, 45 °C; vacuum pressure, 60 Pa	48 h

Note: All treatments used the same pulp dilution ratio, layer thickness, tray format, sample loading, grinding procedure and 60-mesh sieving. The comparison should be interpreted as a practical low-moisture processing comparison rather than a strict equipment-design comparison.

**Table 2:** Physicochemical and functional properties of apricot powders produced by four drying routes.

Physical Properties	HAD	IRD	VPD	VFD
Moisture content (%)	9.85 ± 0.02 ^a^	8.72 ± 0.67 ^b^	8.16 ± 0.19 ^bc^	7.23 ± 0.38 ^c^
Water activity	0.370 ± 0.007 ^a^	0.323 ± 0.008 ^b^	0.270 ± 0.006 ^c^	0.218 ± 0.002 ^d^
Wettability (s)	76.44 ± 1.40 ^a^	62.60 ± 1.29 ^b^	58.21 ± 1.22 ^c^	46.05 ± 1.71 ^d^
Zeta potential (mV)	−21.20 ± 0.23 ^b^	−22.10 ± 0.62 ^ab^	−22.66 ± 1.54 ^ab^	−24.87 ± 1.67 ^a^
Bulk density (g/cm^3^)	0.56 ± 0.01 ^a^	0.54 ± 0.01 ^a^	0.51 ± 0.01 ^b^	0.46 ± 0.01 ^c^
Tapped density (g/cm^3^)	0.61 ± 0.00 ^a^	0.62 ± 0.01 ^a^	0.53 ± 0.00 ^b^	0.51 ± 0.01 ^b^
Particle density (g/cm^3^)	1.21 ± 0.02 ^a^	1.19 ± 0.01 ^ab^	1.17 ± 0.02 ^b^	1.12 ± 0.01 ^c^
Porosity (%)	53.90 ± 1.05 ^c^	54.29 ± 0.18 ^c^	56.48 ± 0.76 ^b^	59.09 ± 0.84 ^a^
D_10_ (μm)	10.40 ± 0.10 ^b^	14.93 ± 0.32 ^a^	9.18 ± 0.12 ^c^	9.56 ± 0.16 ^d^
D_50_ (μm)	80.70 ± 1.11 ^b^	86.33 ± 0.61 ^a^	55.17 ± 0.64 ^c^	52.87 ± 0.84 ^d^
D_90_ (μm)	240.60 ± 4.58 ^a^	203.33 ± 0.58 ^b^	194.67 ± 4.04 ^c^	181.04 ± 2.64 ^d^
D(4,3) (μm)	110.60 ± 2.52 ^a^	98.90 ± 0.44 ^b^	81.23 ± 1.33 ^c^	81.90 ± 2.75 ^c^
Span	2.96 ± 0.04 ^c^	2.18 ± 0.02 ^d^	3.36 ± 0.04 ^a^	3.24 ± 0.01 ^b^
WHC (g/g)	2.42 ± 0.06 ^c^	2.44 ± 0.09 ^c^	3.06 ± 0.08 ^b^	3.63 ± 0.07 ^a^
OHC (g/g)	1.95 ± 0.02 ^c^	2.06 ± 0.02 ^b^	2.10 ± 0.02 ^ab^	2.14 ± 0.01 ^a^

Values are expressed as mean ± standard deviation (*n* = 3). Different lowercase letters within the same row indicate significant differences among treatments or samples (*p* < 0.05). Zeta-potential values were measured in deionized water dispersions without pH adjustment; equilibrium pH was not recorded during the original measurements, and these values should therefore be interpreted as auxiliary comparative dispersion indicators.

**Table 3:** Flowability and handling-related properties of apricot powders produced by four drying routes.

Flowability Index	HAD	IRD	VPD	VFD
Angle of repose (°)	36.23 ± 0.65 ^b^	32.58 ± 0.43 ^c^	28.44 ± 0.34 ^d^	39.61 ± 0.48 ^a^
Hausner ratio	1.18 ± 0.02 ^a^	1.16 ± 0.02 ^a^	1.07 ± 0.01 ^c^	1.11 ± 0.01 ^b^
Carr index (%)	15.51 ± 1.59 ^a^	13.65 ± 1.41 ^a^	6.33 ± 0.66 ^c^	10.23 ± 0.82 ^b^

Values are expressed as mean ± standard deviation (*n* = 3). Different lowercase letters within the same row indicate significant differences among treatments or samples (*p* < 0.05).

**Table 4:** Color attributes of fresh apricot pulp (control) and apricot powders produced by different drying routes.

Attribute	Control	HAD	IRD	VPD	VFD
L*	59.44 ± 0.22 ^a^	51.93 ± 0.65 ^e^	53.51 ± 0.33 ^d^	55.40 ± 0.30 ^c^	58.57 ± 0.48 ^b^
a*	22.52 ± 0.34 ^a^	17.55 ± 0.51 ^b^	17.15 ± 0.35 ^b^	22.19 ± 0.03 ^a^	22.22 ± 0.16 ^a^
b*	58.21 ± 0.54 ^a^	43.85 ± 0.61 ^d^	48.01 ± 0.45 ^c^	56.04 ± 0.11 ^b^	57.46 ± 0.28 ^a^
Chroma	62.42 ± 0.63 ^a^	47.23 ± 0.72 ^d^	50.98 ± 0.54 ^c^	60.24 ± 0.12 ^b^	61.60 ± 0.29 ^a^
Hue angle	68.85 ± 0.11 ^b^	67.94 ± 0.37 ^d^	70.34 ± 0.22 ^a^	68.38 ± 0.01 ^c^	68.86 ± 0.13 ^b^
ΔE	—	16.78 ± 0.90 ^a^	12.63 ± 0.40 ^b^	4.56 ± 0.32 ^c^	1.12 ± 0.47 ^d^

Values are expressed as mean ± standard deviation (*n* = 3). Different lowercase letters within the same row indicate significant differences among treatments or samples (*p* < 0.05). L*, lightness; a*, redness/greenness; b*, yellowness/blueness; ΔE, total color difference.

## Data Availability

The data presented in this study are available from the corresponding authors upon reasonable request. The data are not publicly deposited because the raw replicate files form part of an ongoing apricot-powder processing dataset.

## Supplementary Materials

The following supporting information can be downloaded at https://www.mdpi.com/article/10.3390/foods15142455/s1, Figure S1: X-ray diffraction patterns of apricot powders produced by different drying routes; Figure S2: FTIR spectra of apricot powders produced by different drying routes in the range of 4000–400 cm^−1^.

## Abbreviations

The following abbreviations are used in this manuscript:

AAC	Ascorbic acid content
ANOVA	Analysis of variance
aw	Water activity
BD	Bulk density
CI	Carr index
CIELAB	Commission Internationale de l’Éclairage L*a*b* color space
D(4,3)	Volume-weighted mean diameter
D_10_	Particle diameter below which 10% of the particle volume is found
D_50_	Median particle diameter
D_90_	Particle diameter below which 90% of the particle volume is found
FRAP	Ferric reducing antioxidant power
FTIR	Fourier transform infrared spectroscopy
GC–IMS	Gas chromatography–ion mobility spectrometry
GC–MS	Gas chromatography–mass spectrometry
HAD	Hot-air drying
HR	Hausner ratio
IRD	Infrared drying
OHC	Oil-holding capacity
PCA	Principal component analysis
PD	Particle density
RSC	Reducing sugar content
SD	Standard deviation
SEM	Scanning electron microscopy
TAE	Tannic acid equivalent
TCC	Total carotenoid content
TD	Tapped density
TPC	Total phenolic content
TSC	Total sugar content
VFD	Vacuum freeze drying
VPD	Vacuum-pulsed drying
WHC	Water-holding capacity
XRD	X-ray diffraction
ΔE	Total color difference

## Appendix A

**Table A1:** Sensor array and main response characteristics of the PEN3.5 electronic nose.

Array Number	Sensor Name	Representative Substance Categories	Performance Description
1	W1C	Aromatic	Aromatic constituents, benzene
2	W5S	Broadrange	High sensitivity and sensitive to nitrogen oxides
3	W3C	Aromatic	Sensitive aroma, ammonia
4	W6S	Hydrogen	Mainly selective for hydrides
5	W5C	Arom-aliph	Short-chain alkane aromatic component
6	W1S	Broad-methane	Sensitive to methyl
7	W1W	Sulphur-organic	Sensitive to sulfides
8	W2S	Broad-alcohol	Sensitive to alcohols, aldehydes and ketones
9	W2W	Sulph-chlor	Aromatic ingredients, sensitive to organic sulfides
10	W3S	Methane-aliph	Sensitive to long-chain alkanes
