# Supplementary material for: Drying Route Influences Matrix Organization, Reconstitution, Flowability and Selected Phytochemical Indicators of Apricot Powder
Source: Foods. 2026 Jul 10;15(14):2455. doi: 10.3390/foods15142455 (PMC13407703; doi:10.3390/foods15142455)
Supplement: Supplementary file 1 [file foods-15-02455-s001.zip › foods-4386266-supplementary.pdf]

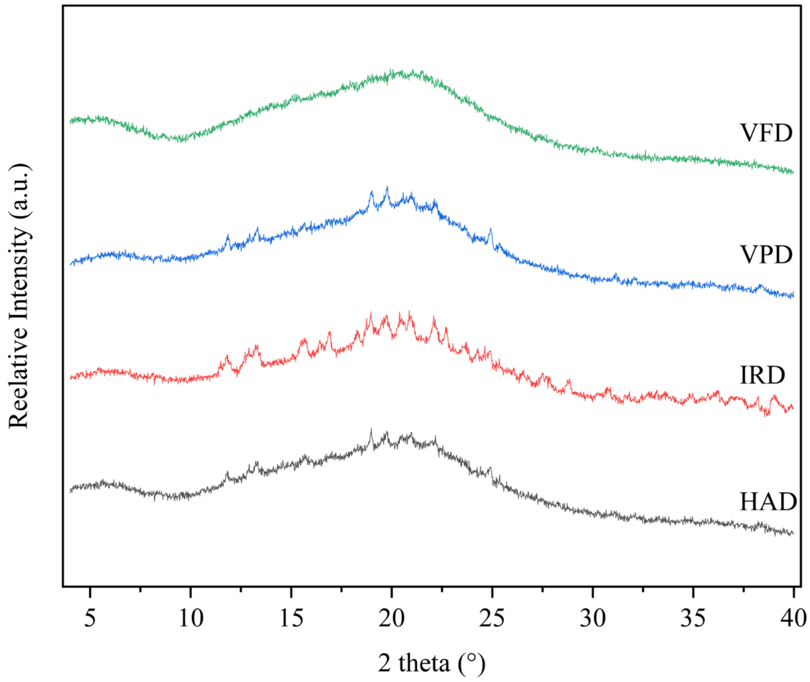

Figure S1. X-ray diffraction patterns of apricot powders produced by different drying routes.

XRD patterns provided complementary information on drying-route-dependent differences in molecular ordering (Figure S1). All apricot powders showed broad diffraction features rather than dominant sharp crystalline reflections, indicating that the powder matrix was mainly amorphous or weakly ordered after drying and grinding. Such differences in short-range ordering may accompany changes in matrix packing and water interaction. However, XRD does not directly measure pore accessibility, capillary structure, particle morphology, wettability or flowability. These differences were generally consistent with the treatment-dependent density and calculated porosity trends shown in Table 2. Nevertheless, XRD alone cannot quantify matrix compactness, pore connectivity or the mechanisms governing reconstitution and flowability.

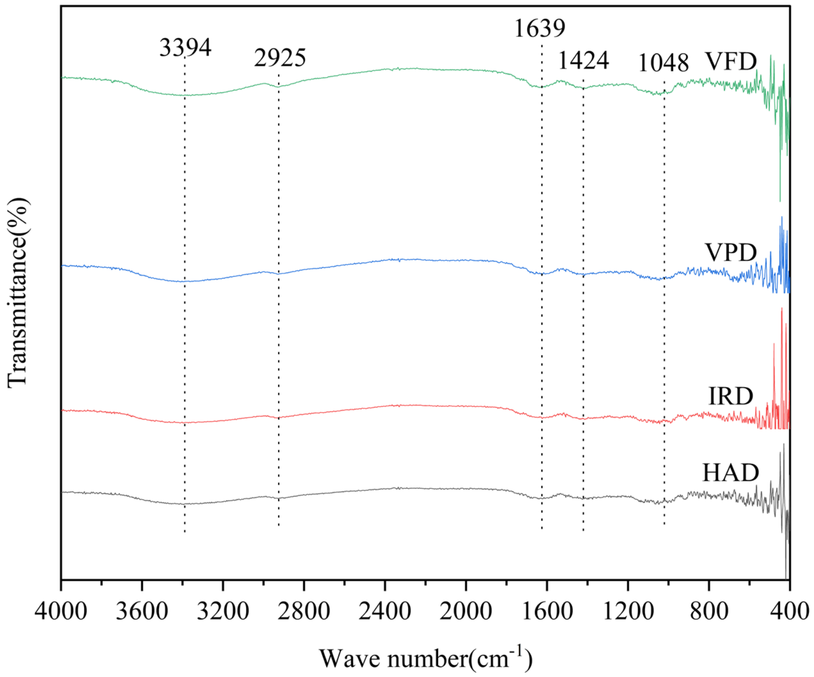

Figure S2. FTIR spectra of apricot powders produced by different drying routes in the range of 4000–400 cm^{-1}.

FTIR spectra further indicated that the major functional groups of apricot powders were generally retained after different drying treatments (Figure S2). The broad band around 3394 cm^{-1} was associated with O–H stretching, the band near 2925 cm^{-1} with C–H stretching, the absorption around 1639 cm^{-1} with bound water or carbonyl-related vibrations, and the bands near 1424 and 1048 cm^{-1} with carbohydrate- and polysaccharide-related vibrations. The similarity of the main absorption bands indicated that no obvious new functional groups were generated by the drying routes. Variations in band intensity may reflect treatment-dependent differences in water association and hydrogen-bonding environments, although band intensity can also be affected by sample preparation and pellet characteristics. The general similarity of the spectra indicates that the major differences in hydration and handling behavior were unlikely to result from the formation of new dominant functional groups. They were more plausibly associated with physical organization, residual moisture and particle characteristics, although FTIR alone cannot establish these mechanisms.
